# Erysipelas on surgical scar: a case report

**DOI:** 10.11604/pamj.2020.35.30.17551

**Published:** 2020-02-07

**Authors:** Asma Korbi, Ahmed Hajji, Hela Dahmani, Farouk Ennaceur, Haifa Bergaoui, Awatef Hajjaji, Raja Faleh

**Affiliations:** 1 Department of Gynecology and Obstetrics at the University Hospital of Monastir, Monastir, Tunisia

**Keywords:** Case report, erysipelas, patey scar, post-operative

## Abstract

Erysipelas is a non-necrotizing acute dermal hypodermatitis most often of streptococcal origin. It most often affects the lower limbs. Erysipelas on surgical scar has been rarely reported in the literature. Few cases have been published since the first descriptions of this pathological entity by Baddour *et al* in 1982. We report the case of a 47-year-old patient. Operated for right breast mucinous carcinoma, she had neo-adjuvant chemotherapy followed by a surgical treatment (Patey) which occured without incident. The evolution was marked by the appearance after 11 months of the intervention of an Erysipelas on Patey scar. The patient was put on cefazol for 7 days intravenously injectable. The evolution was marked by the complete disappearance of the rash and the edema.

## Introduction

Erysipelas is a non-necrotizing acute dermal hypodermatitis most often of streptococcal origin [1]. It most often affects the lower limbs being favored by the presence of an entryway more often cutaneous [2]. Lymphedema and obesity are the main risk factors. In the literature, this pathological entity has rarely been described on a postoperative cutaneous scar [3]. We report in this work the case of a woman who presented an erysipelas on patey scar. The purpose of our work is to specify the epidemiological, clinical and therapeutic characteristics of the post-operative erysiple and in particular on patey scar.

## Patient and observation

**Figure 1:**
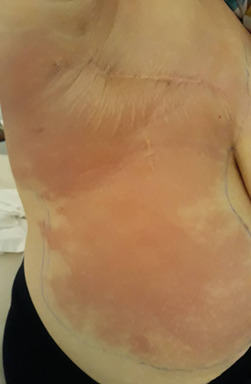
Erysipelas on patey scar

We report the case of a 47-year-old patient with a medical history of codeine allergy, surgical appendectomy. She consulted for bilateral mastodynia. The examination found a 3 cm mass at the suspicious QSID, no associated axillary ADP. A mammography echo was requested showing three masses of QSID requiring histological verification especially the pre-chest mass of 15mm. Right breast grafted ACR 4, left breast graft ACR 1.A biopsy was made confirming the diagnosis of right breast mucinous carcinoma of carcinoma having as histo-protected grade modified SBR II. No overexpression of the HER 2 protein. An extension assessment (an abdominal ultrasound, a chest X-ray with a bone scan) does not show a secondary-level lesion. She had neo-adjuvant chemotherapy followed by a surgical treatment (Patey) which occured without incident. The evolution was marked by the appearance after 11 months of the intervention in a febrile context of a warm erythematous rash, painful and oedematous carrying the right patey scar and reaching the umbilicus, with purpuric lesions without crepitants or sensory disturbances or fistulization, without lymphoedema or associated ADP (Figure 1).

The biological examination shows an increase in activated protein C at 159 mg/l, white blood cells at 27,000, normal fasting glucose. Ultrasound does not show an abscess or an underlying collection. As soon as the diagnosis of erysipelas on patey scar was retained, the patient was put on cefazol 2gr * 3 / day for 7 days intravenously injectable and rest. A clinico-biological improvement was noted (durable apyrexia with decrease of the CRP) hence the passage to the oral way. The evolution was marked by the complete disappearance of the rash and the edema.

## Discussion

Erysipelas on surgical scar has been rarely reported in the literature. Indeed, few cases have been published since the first descriptions of this pathological entity by Baddour *et al* [1] in 1982. In this respect, this author treated in 1997 a series of 5 cardiac patients, having developed an erysipelas of the lower limbs. On veinectomy scars for coronary artery bypass grafting. In the same circumstances of comorbidity (heart disease), but for a larger series, Karakas *et al* [2] had published in 2002 a work that focused on 31 cases. In traumatological and orthopedic surgery, we could find in the archives of the literature only the results of the work of Dhrif *et al* [3] having collected, on his retrospective study carried out between 1999 and 2003, 3 cases occurred in the suites of a prosthesis implantation Osteoarticular disease in patients with a mean age of 61 years, one of whom had chronic venous failure. On the pathophysiological level, most authors consider the operative scar as a source of alteration of lymphatic drainage and consequently a risk factor for the occurrence of erysipelas of the leg. The incidence is all the more important in the presence of the other classic factors namely diabetes, obesity and lymphoedema that were missing in our patient, in whom the appearance of erysipelas seems to be due to the concomitant combination of a cutaneous entryway and a defect of the drainage at the level of the local lymphatic microcirculation, destroyed during surgery during the surgical access of this fragile zone. In the literature, the appearance of erysipelas can occur both early and late compared to the date of the scar. The delay varies between 2 months to 2 years in the series of Baddour [1], and varies in that of Hattab [4] between 8 months to 26 years. These authors relate the early onset of erysipelas in patients relative to others, the coexistence of multiple risk factors, and the comorbidity of elderly patients. In our context, the erythematous rash occurred 11 months after surgery, which is an early delay that we explain in the absence of conventional risk factors, a lack of lymphatic drainage. Clinically, erysipelas on an operative scar has the same characteristics as erysipelas on healthy skin [1, 5, 6]. It is a hot painful inflammatory erythematous rash encompassing the cutaneous scar. Lymphadenopathies are present in about 30% of cases and pre-existing lymphoedema is reported with an incidence of 20% [4, 5, 7]. In our patient, the clinical presentation was typical of the association of an erythema rash with a biological inflammatory syndrome. In the literature, the antibiotic treatment of erysipelas on an operative scar will require neither a longer duration nor larger doses of penicillin. The average duration of antibiotic therapy is 15 days in simple forms. It is longer for up to 21 days in subjects with underlying comorbidities.

## Conclusion

Erysipelas on postoperative scar is a complication rarely described in oncological surgery. It has the same clinical and therapeutic characteristics as erysipelas on healthy skin. Its frequency is explained by the presence of the secondary lymoedema during axillary dissection. As a result, the primary prevention of this pathological entity, which involves simple measures, must be the subject of a systematic prescription of postoperative physiotherapy.
